# A plain language summary of the perspectives of women who were interviewed about their experiences with uterine fibroids

**DOI:** 10.57264/cer-2023-0195

**Published:** 2024-05-28

**Authors:** Elke Hunsche, Viatcheslav Rakov, Kayla Scippa, Brooke Witherspoon, Laura McKain

**Affiliations:** 1 Sumitomo Pharma Switzerland GmbH, Basel, Switzerland; 2 Endpoint Outcomes, Long Beach, CA, USA; 3 Lumanity Patient-Centered Outcomes, Boston, MA, USA; 4 Myovant Sciences, Inc., Brisbane, CA, USA

**Keywords:** heavy menstrual bleeding, lay summary, patient perspective, plain language summary, uterine fibroids, women's health

## Abstract

**What is this summary about?:**

This summary describes what researchers learned during interviews of women with uterine fibroids and heavy menstrual bleeding (or period bleeding). At this time, little is known about how women perceive the impact of uterine fibroids on their lives and more information is needed. The goal of this study was to provide new information about the symptoms women have and how these symptoms affect their everyday lives. These interviews were done to better understand how uterine fibroid symptoms affect the lives of women in their own words.

**What were the results?:**

Thirty women from the United States, who had completed a clinical trial for a new treatment for heavy menstrual bleeding and uterine fibroids, agreed to be interviewed. The women described what their experiences with uterine fibroids were and the impact these experiences with uterine fibroids had on their lives before participating in the clinical trial. The most common symptoms of uterine fibroids the women described were heavy bleeding with their menstrual periods, pain in the pelvis or groin area, the passing of blood clots, and anemia (or low hemoglobin in red blood cells). Women said their symptoms affected them physically, emotionally, socially, and financially. They also said their symptoms made it hard to do daily activities, sleep, have a sex life, and go to work or school.

**What do the results mean?:**

Women who have heavy menstrual bleeding and uterine fibroids experience various uterine fibroid symptoms, and these symptoms affect most parts of the their lives.

**This is an abstract of the Plain Language Summary of Publication article.**

To read the full Plain Language Summary of this article, click here to view the PDF.

Link to original article here

